# Dynamic interventions to control COVID-19 pandemic: a multivariate prediction modelling study comparing 16 worldwide countries

**DOI:** 10.1007/s10654-020-00649-w

**Published:** 2020-05-19

**Authors:** Rajiv Chowdhury, Kevin Heng, Md Shajedur Rahman Shawon, Gabriel Goh, Daisy Okonofua, Carolina Ochoa-Rosales, Valentina Gonzalez-Jaramillo, Abbas Bhuiya, Daniel Reidpath, Shamini Prathapan, Sara Shahzad, Christian L. Althaus, Nathalia Gonzalez-Jaramillo, Oscar H. Franco, The Global Dynamic Interventions Strategies for COVID-19 Collaborative Group

**Affiliations:** 1 Department of Public Health and Primary Care, School of Clinical Medicine, University of Cambridge, Cambridge, UK; 2 Center for Space and Habitability, University of Bern, Bern, Switzerland; 3 Department of Physics, Astronomy and Astrophysics Group, University of Warwick, Coventry, UK; 4 Centre for Big Data Research in Health, University of New South Wales, Sydney, Australia; 5 OpenAI Artificial Intelligence Research Laboratory, San Francisco, CA USA; 6 Department of Epidemiology, Erasmus MC - University Medical Center Rotterdam, Rotterdam, The Netherlands; 7 Centro de Vida Saludable, Universidad de Concepción, Concepción, Chile; 8 Institute of Social and Preventive Medicine, University of Bern, Bern, Switzerland; 9 Independent health and population researcher, Dhaka, Bangladesh; 10 International Centre for Diarrhoeal Disease Research, Dhaka, Bangladesh; 11 Department of Community Medicine, University of Sri Jayewardenepura, Colombo, Sri Lanka

**Keywords:** COVID-19, Prediction modelling, Dynamic interventions, Infectious disease, Epidemiology

## Abstract

To date, non-pharmacological interventions (NPI) have been the mainstay for controlling the coronavirus disease-2019 (COVID-19) pandemic. While NPIs are effective in preventing health systems overload, these long-term measures are likely to have significant adverse economic consequences. Therefore, many countries are currently considering to lift the NPIs—increasing the likelihood of disease resurgence. In this regard, dynamic NPIs, with intervals of relaxed social distancing, may provide a more suitable alternative. However, the ideal frequency and duration of intermittent NPIs, and the ideal “break” when interventions can be temporarily relaxed, remain uncertain, especially in resource-poor settings. We employed a multivariate prediction model, based on up-to-date transmission and clinical parameters, to simulate outbreak trajectories in 16 countries, from diverse regions and economic categories. In each country, we then modelled the impacts on intensive care unit (ICU) admissions and deaths over an 18-month period for following scenarios: (1) no intervention, (2) consecutive cycles of mitigation measures followed by a relaxation period, and (3) consecutive cycles of suppression measures followed by a relaxation period. We defined these dynamic interventions based on reduction of the mean reproduction number during each cycle, assuming a basic reproduction number (*R*_0_) of 2.2 for no intervention, and subsequent effective reproduction numbers (*R*) of 0.8 and 0.5 for illustrative dynamic mitigation and suppression interventions, respectively. We found that dynamic cycles of 50-day mitigation followed by a 30-day relaxation reduced transmission, however, were unsuccessful in lowering ICU hospitalizations below manageable limits. By contrast, dynamic cycles of 50-day suppression followed by a 30-day relaxation kept the ICU demands below the national capacities. Additionally, we estimated that a significant number of new infections and deaths, especially in resource-poor countries, would be averted if these dynamic suppression measures were kept in place over an 18-month period. This multi-country analysis demonstrates that intermittent reductions of *R* below 1 through a potential combination of suppression interventions and relaxation can be an effective strategy for COVID-19 pandemic control. Such a “schedule” of social distancing might be particularly relevant to low-income countries, where a single, prolonged suppression intervention is unsustainable. Efficient implementation of dynamic suppression interventions, therefore, confers a pragmatic option to: (1) prevent critical care overload and deaths, (2) gain time to develop preventive and clinical measures, and (3) reduce economic hardship globally.

**Electronic supplementary material:**

The online version of this article (10.1007/s10654-020-00649-w) contains supplementary material, which is available to authorized users.

## Introduction

Coronavirus disease 2019 (COVID-19) pandemic has imposed an unprecedented challenge to global healthcare systems, societies, and governments [1]. As of May 16, 2020, the severe acute respiratory syndrome coronavirus-2 (SARS-CoV-2, causative pathogen for COVID-19) has been detected in every country, with more than 4.6 million confirmed cases and a death toll exceeding 300,000 worldwide [2]. Furthermore, recent pandemic model projections estimate that COVID-19 could result in ~ 40 million deaths globally this year, if no interventions are implemented [3]. To date, in the absence of efficacious pharmaceutical measures for prevention or treatment, the principal strategy to control COVID-19 has focused on community-based, non-pharmaceutical interventions (NPIs) [4]. These NPIs typically include a package of mitigation and suppression measures (e.g., case-based isolation, shielding of vulnerable groups, school closures, restricting public events and lockdowns), that aim to minimize person-to-person transmissions of SARS-CoV-2 through social distancing [5].

While NPIs are effective (e.g., in blunting the peak of the epidemic, preventing health systems overload and reducing incidence) [4, 6, 7], these long-term measures are also associated with significant unemployment, economic hardship and social disruption (with surveys from resource-poor settings showing an average fall in income by 70% and consumption expenditure by 30%) [8]. There is a growing concern whether these prolonged interventions are sustainable given the widespread disparities in economic resilience and health sector capacities globally [9]. As a result, many countries worldwide are currently considering to lift the lockdowns—increasing the likelihood of disease resurgence. In this regard, dynamic NPIs with intervals of relaxed social distancing, may serve as a realistic alternative to achieve the NPI goals, with minimal adverse socioeconomic consequences. However, it remains unclear (1) what should be the frequency and duration of such dynamic NPIs, (2) what should be the ideal “break” when interventions can be relaxed temporarily before case numbers resurge, and (3) which dynamic NPI strategy should be adapted globally across regions with diverse health and economic infrastructures. Addressing these issues is essential to devise feasible, context-specific policies to prevent collapse of healthcare systems, reduce premature deaths and minimize detrimental impacts on national economies associated with prolonged continuous NPIs.

To address these uncertainties, we have employed a transmission dynamic model comparing sixteen countries that vary in setting and income groupings. Our key aims were to: (1) calculate age-standardized estimates of case-severity and fatality in included countries; (2) estimate the impact of an uncontrolled course of the pandemic in each country, given the current resources of their health systems (counterfactual), (3) compare continuous versus intermittent combinations of mitigation/suppression and relaxation strategies, over an 18-month period (i.e., optimistic timeline for an efficacious vaccine to be developed [10]); and (4) identify strategies that help keep the number of projected cases requiring critical care within a manageable limit, while also considering a feasible duration of these interventions.

## Methods

This study was conducted according to the to the TRIPOD reporting guideline [11] for prediction modelling studies (Supplementary Appendix 1).

### Study design, source of data and study settings

We have employed a multivariate prediction model to describe COVID-19 transmission dynamics under various NPIs. Since the distributions of age and underlying co-morbidities may differ importantly by country, region and economic status [4] we have hypothesised that the predicted mortality impacts for NPI strategies will differ importantly. Therefore, for this current study, we have considered several circumstances. First, we used age-standardized clinical dynamic estimates to model the epidemic trajectories in 16 different countries (which comprise roughly a quarter of the global population), by accessing available country-specific age structure data. Second, we selected these countries from diverse geographical regions: Western Europe (The Netherlands, Belgium), South America (Chile, Colombia), North America (Mexico), Africa (South Africa, Nigeria, Ethiopia, Tanzania, Uganda), South Asia (India, Bangladesh, Pakistan Sri Lanka), West Asia (Yemen), and the Pacific (Australia). Third, these countries also represent all income categories equally, as defined by the World Bank [12]: four countries in every high (HIC), higher-middle (HMIC), lower-middle (LMIC) and low income (LIC) groups, respectively.

### Intervention scenarios, predictors and outcomes

We considered case isolation at home, voluntary home quarantine, closure of schools and universities, and social distancing of the entire population as physical distancing measures. We defined the study interventions scenarios based on reduction of the reproduction number during the duration of intervention (*R*). For this, we assumed a basic reproduction number [13] (*R*_0_, the average number of secondary infections arising from a typical single infection in a completely susceptible population) of 2.2 for uncontrolled spread of COVID-19, and effective reproduction numbers (*R*, average number of secondary cases per infectious case in presence of control measures and a partially immune population) of 0.8 and 0.5 for mitigation and suppression interventions, respectively. These assumptions were based on recent work by Jarvis et al. [14] who reported a 73% reduction in the average daily number of contacts observed per participant for physical distancing measures. This corresponded to a pre-intervention *R*_0_ value of 2.6 to reduce to a post-intervention *R* value of 0.62 (95% confidence interval: 0.37–0.89) following strict suppression measures. Even though the exact relationship between changes in the number of social contacts and *R*_0_ remains unclear, we used these findings as the rationale to calculate our study’s effective *R* values of 0.5 and 0.8 for the interventions. These numbers are in agreement with recent estimates for several European countries and arguably reflect the expected effects of a somewhat relaxed and more stringent lockdown [15].

Based on this approach, or each country, the following intervention scenarios were considered: (1) no intervention (i.e., counterfactual scenario), (2) consecutive cycles of mitigation (a combination of measures, such as general social distancing measures, hygiene rules, case-based isolation, shielding of vulnerable groups, school closures or restricting of large public events; target *R* = 0.8), followed by a relaxation period (comprising of case-based home isolation of positive cases and shielding of vulnerable groups), (3) consecutive cycles of suppression (additional measures of strict physical distancing, including lockdowns; target *R* = 0.5) followed by a relaxation period (as defined above), and (4) a continuous suppression measure with no relaxation.

In the absence of intervention, the assumed parameters for transmission dynamics yielded a characteristic rise-and-fall timescale of infections of about 50 days, which we set to be the illustrative duration of intervention. Choosing a slightly longer period (e.g. 60 days) yielded similar outcomes. The duration of breaks between interventions needs to be less than the intervention period for the interventions to be effective; therefore, we set the break duration to be 30 days. When to intervene was determined by the initial fraction of the population that was infected. For example, if the fraction was on the order of 1 part in 10,000 (or more), we set the initiation point for the intervention at Day 20. However, if the fraction was on the order of 1 part in 100,000 to 1 million, we set the initiation point as Day 30. Similarly, if the fraction was on the order of 1 part in 10 million, we set this at Day 50. Changes in the initial fraction simply shift the curves back and forth in time without altering their shapes.

For each country, the outcomes of interest were (1) the number requiring intensive care unit (ICU) beds (primary outcome); and (2) total number of hospitalizations and deaths (secondary outcome), by different scenarios of NPIs, and within a time horizon of 18 months. We prioritized ICU care needs as the main outcome since this healthcare component is in short supply in many resource-limited settings, and therefore, is a major determinant for adverse COVID-19 outcomes.

### Statistical methods for model calibration and age-standardization

The analyses were based on a standard susceptible-exposed-infected-recovered (SEIR) compartmental model [16] to describe the transmission of SARS-CoV-2 in 16 countries under various NPI scenarios. The model considered additional compartments for hospitalization and ICU demand. Susceptible individuals *S* are infected by infectious individuals *I* at a rate *β*. After an incubation period of 1/*σ* = 5·2 days [17], exposed individuals *E* becomes infectious *I*, and either clear the infection at a rate *γ* or progress to severe infection *P* with probability *f*_*P*_. The infectious period is taken to be 1/*γ* = 2·3 days, corresponding to a serial interval and generation time of 1/*σ* + 1/*γ* = 7·5 days [17]. The quantity *f*_*P*_ is the proportion of infections that require hospitalization, for which we obtained age-specific estimates from a recent analysis of COVID-19 cases in China [18].

We applied these age-specific estimates to each individual country’s population to get country-specific age-standardized proportion of infections that require hospitalization. We considered the delay between severe infection and hospitalization is 1/*ω* = 2·7 days [4]. Severely infected individuals *P* enter the hospital as *H*, after which they either leave the hospital at a rate *κ* or enter the ICU with probability *f*_*U*_. Age-stratified proportions of hospitalized cases requiring ICU care (*f*_*U*_) were based on the Imperial College COVID-19 Response Team’s Report [4], and then standardized according to each country’s population age structure. The quantity 1/*κ* is the duration of non-ICU hospital stays, which we considered 8 days [4]. Patients *U* stay in ICU for 1/*δ* = 8 days [4], after which a fraction of them die (*f*_*D*_). The age-specific infectious fatality rate (IFR) were obtained from Verity et al. [18]. Those were subsequently applied to individual country’s population to get country-specific age-standardized IFRs (Supplementary Tables S1–S16). IFR is the product of *f*_*P*_, *f*_*U*_, and *f*_*D*_. The basic reproduction number is *R*_0_ = *βN*/*γ* = 2·2 [17, 19, 20], with *N* being the total population size of the country. The set of coupled ordinary differential equations that underpin our model are presented in the Box 1. These equations in the SEIR model were solved numerically using the *solve_ivp* package in the Python programming language suite [21]; plots were created using the *matplotlib* graphics package [22].

**Box 1:** Equations used in SEIR compartmental model

$$\frac{dS}{dt}= -\beta IS,$$
$$\frac{dE}{dt}= \beta IS- \sigma E,$$
$$\frac{dI}{dt}= \sigma E-\gamma I,$$
$$\frac{dP}{dt}= {f}_{P}\gamma I-\omega P,$$
$$\frac{dH}{dt}= \omega P-\kappa H,$$
$$\frac{dU}{dt}= {f}_{U}\kappa H-\delta U,$$
$$\frac{dR}{dt}=\left(1-{f}_{P}\right)\gamma I+\left(1-{f}_{U}\right)\kappa H+(1-{f}_{D})\delta U,$$
$$\frac{dD}{dt}= {f}_{D}\delta U.$$

## Results

### Country-specific characteristics and clinical dynamics

*Demographic and health system-related characteristics* Table 1 presents a summary of the demographic and health system-related characteristics for the included countries, grouped by their respective income levels. Briefly, the countries varied in population size (ranging from 11,539,326 in Belgium to 1,366,417,755 in India). The first cases were identified in a much later date in the LICs (~ late February–early March, 2020) compared to HIC countries such as Australia, the Netherlands and Belgium. Additionally, there were significant differences across countries with respect to healthcare infrastructure. For example, in the majority of LICs and LMICs, available hospital and ICU beds were < 1 bed per 1000 population and < 1 bed per 100,000 population, respectively (Table 1).

**Table 1:** Key demographic and health system-related characteristics of the 16 included countries

	Size of population	Number of initial infections (as of 1 April 2020)^a^	Date of first case	Hospital beds per 1000 population^b^	Total hospital beds	Total ICU beds^c^	ICU beds per 100,000 population
High-income							
Australia	25,203,200	9618	25 January 2020	3.8	95,772	2200	8.7
Belgium	11,539,326	11,899	04 February 2020	6.2	71,544	1900	16.5
Chile	18,952,035	2449	03 March 2020	2.2	41,694	1000	5.3
The Netherlands	17,097,123	11,750	27 February 2020	4.7	80,356	1150	6.7
Upper-middle income							
Colombia	50,339,443	702	06 March 2020	1.5	75,509	5600	11.1
Mexico	127,575,528	993	28 February 2020	1.5	191,363	3000	2.4
South Africa	58,558,267	1326	05 March 2020	2.5	146,396	1500	2.6
Sri Lanka	21,323,734	112	27 January 2020	3.6	76,765	519	2.4
Lower-middle income							
Bangladesh	163,046,173	49	08 March 2020	0.8	130,437	1174	0.7
India	1,366,417,755	1251	30 January 2020	0.9	1,229,776	29,997	2.2
Nigeria	200,963,603	111	27 February 2020	0.5	100,482	128	0.1
Pakistan	216,565,317	1865	26 February 2020	0.6	129,939	3142	1.5
Low-income							
Afghanistan	38,041,757	166	24 February 2020	0.5	19,021	100	0.3
Burkina Faso	20,321,383	246	09 March 2020	0.4	8,129	50	0.2
Tanzania	58,005,461	19	16 March 2020	0.7	40,604	38	0.1
Uganda	44,269,587	33	20 March 2020	0.5	22,135	55	0.1

*ICU* intensive care unit

^a^Taken from various country-specific reports

^b^Taken from The World Bank Data on hospital bed [23]

^c^Taken from various country-specific reports

*Age-standardized estimates of case-severity and fatality* Table 2 summarizes various COVID-19 relevant clinical dynamics estimated for each of the 16 included countries. Briefly, proportion of infected individuals who require hospitalization ranged from 1.61% in Uganda to 6.12% in the Netherlands, with higher proportions observed in HIC and UMICs compared to the other country categories. This pattern was similar for the proportion of hospitalized cases requiring critical care (Table 2). IFR estimates were significantly higher in the HICs, compared to LMIC and LICs (range 0.17 in Burkina Faso to 1.13 in Belgium).

**Table 2:** Age-standardised estimates for case severity and fatality of COVID-19 for 16 included countries

	Proportion of infected individuals hospitalised^a^ (%)	Proportion of hospitalised cases requiring critical care^b^ (%)	Proportion of individuals requiring critical care die^c^ (%)	Infection fatality ratio (*IFR*)^d^ (%)
High-income				
Australia	5.34	29.3	59.6	0.93
Belgium	6.01	31.5	59.6	1.13
Chile	4.69	25.8	59.5	0.72
The Netherlands	6.12	30.6	59.6	1.12
Upper-middle income				
Colombia	3.93	23.3	59.4	0.54
Mexico	3.57	22.3	59.4	0.47
South Africa	3.09	19.1	59.2	0.35
Sri Lanka	4.38	24.2	59.5	0.63
Lower-middle income				
Bangladesh	3.10	19.6	59.3	0.36
India	3.35	20.3	59.3	0.41
Nigeria	1.96	16.3	59.1	0.19
Pakistan	2.55	19.0	59.2	0.29
Low-income				
Afghanistan	1.86	16.4	59.1	0.18
Burkina Faso	1.81	16.0	59.0	0.17
Tanzania	1.90	16.3	59.0	0.18
Uganda	1.61	15.1	58.9	0.15

All estimates are standardised according to the age structure of the respective country

^a^Age-specific proportions of infected individuals hospitalised were taken from Verity et al. [18]. These proportions were adjusted for under-ascertainment and corrected for demography. We assumed that cases defined as severe would be hospitalised

^b^Age-specific proportions of hospitalised cases requiring critical care were taken from Imperial COVID-19 Response Team Report [4]

^c^Age-specific proportions of individuals requiring critical care die were calculated by dividing the IFRs with proportions of infected individuals hospitalised and proportions of hospitalised cases requiring critical care

^d^Age-specific *IFR*s were taken from Verity et al. [18]

### Model development and predicted impact of the interventions

**Fig. 1:**
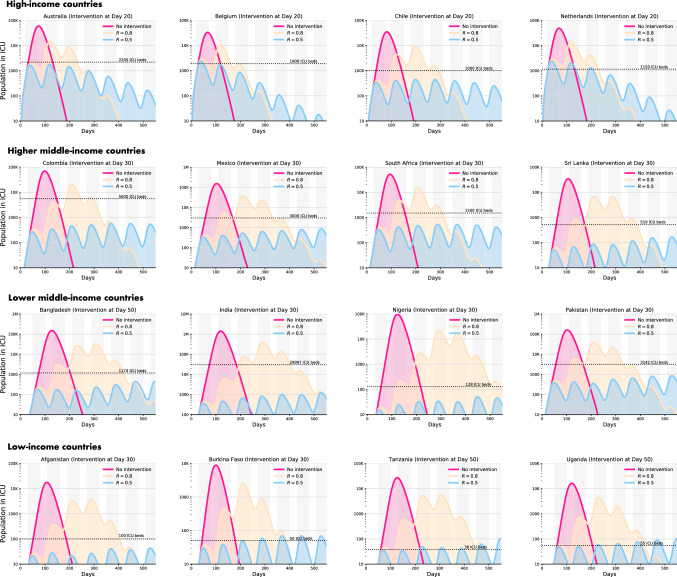
Impact of dynamic interventions and relaxation on ICU beds requirement in 16 countries over an 18-month period

* Impact of uncontrolled or no intervention scenario* In the unlikely scenario of no NPI, the number of cases requiring ICU care would exceed the available capacity significantly for every single country (Fig. 1). This unmitigated scenario, in aggregate, would also result in 7,840,444 deaths in all 16 countries. This estimate would have been equivalent to approximately 46% of all deaths recorded in these countries in 2017. Additionally, an uncontrolled epidemic would predict 583,738 total deaths in the HIC, 1,026,361 deaths in the HMIC, 6,000,220 deaths in the LMIC, and 230,125 deaths in the LIC settings. The majority of these deaths will occur in India, proportionate to the large population of this country. Under this scenario, the duration of the epidemic will last nearly 200 days in the majority of the included countries (Fig. 1).

*Comparing impacts of dynamic cycles of mitigation/suppression and relaxation* Our models predict that simultaneous cycles of 50-day mitigation intervention followed by a 30-day relaxation would likely to reduce the effective reproduction number *R* to 0.8 in all countries. However, this rolling mitigation measure was insufficient to keep the number of patients requiring healthcare below the available critical care capacity (Fig. 1). In this NPI scenario, the duration of pandemic appeared approximately 12 months in the HIC, and was close to 18 months in the other settings. Additionally, dynamic mitigation interventions were effective at the first 3 months for all the countries, but after the first relaxation, the pandemic would exceed the hospital capacity in all the countries and would result in 3,534,793 deaths. By contrast, we found that dynamic cycles of 50-day suppression followed by a 30-day relaxation, aimed at reducing the effective *R* to 0.5, were suitable for all settings to keep ICU demand within national capacity (Fig. 1). Since more individuals remain susceptible at the end of each cycle of suppression and relaxation, such approach would result in a longer pandemic, beyond 18 months in all countries; however, global mortality would drop to 131,643 during that period (Fig. 1).

Estimated impacts of dynamic mitigation and suppression strategies on new infections, hospitalisations and deaths in all 16 countries have been summarised in Table 3. Briefly, the numbers of new infections per day (during the peak of epidemic) were significantly higher for all countries in no and dynamic mitigation intervention scenarios. Both new infections and ICU bed requirements per day (during the peak of epidemic) were significantly lower, especially for low-income settings, for dynamic suppression and relaxation strategy (Table 3). For dynamic mitigation strategies, mortality estimates were 266,835 in HICs, 463,499 in HMICs, 2,700,162 in LMICs, were and 104,297 in LICs. The corresponding estimates for the dynamic suppression strategies were markedly lower: 63,166 in HICs, 32,419 in HMICs, 32,210 in LMICs and 3,848 in LICs (Table 3).

**Table 3:** The estimated impacts of various interventions on COVID-19 outcomes in 16 countries

Countries and income categories	Uncontrolled, no intervention scenario			Intermittent cycles of mitigation and relaxation (Effective *R* = 0.8)			Intermittent cycles of suppression and relaxation (Effective *R* = 0.5)		
	New infections/day during the peak	ICU bed needs/day during the peak	No. of total deaths over 18 months	New infections/day during the peak	ICU bed needs/day during the peak	No. of total deaths over 18 months	New infections/day during the peak	ICU bed needs/day during the peak	No. of total deaths over 18 months
High-income									
Australia	1,434,638	59,803	197,746	418,643	14,798	89,091	54,748	1734	19,996
Belgium	657,883	33,213	109,785	253,150	10,674	51,151	63,135	2404	15,846
Chile	1,078,061	34,818	115,060	357,316	9716	53,210	18,351	450	7505
The Netherlands	973,779	48,724	161,147	354,373	14,831	73,383	63,412	2395	19,819
Upper-middle income									
Colombia	2,862,000	69,878	230,682	988,841	20,225	104,040	30,730	570	9239
Mexico	7,253,642	154,507	509,794	2,082,308	37,598	228,879	53,308	863	12,047
South Africa	3,329,773	52,421	172,416	1,189,739	15,674	79,091	44,377	531	9094
Sri Lanka	1,212,623	34,335	113,469	282,813	6876	51,489	7875	170	2039
Lower-middle income									
Bangladesh	9,270,170	150,503	495,420	2,427,104	33,631	226,700	36,597	452	4908
India	77,698,771	1,414,384	4,660,013	26,185,375	399,982	2,093,893	87,558	1211	15,379
Nigeria	11,426,973	97,411	319,598	2,944,575	21,424	144,049	7894	51	659
Pakistan	12,316,925	159,636	525,189	3,653,682	40,072	235,520	86,084	848	11,264
Low-income									
Afghanistan	2,163,088	17,640	57,851	550,669	3839	26,401	6989	43	614
Burkina Faso	1,155,479	8918	29,228	388,909	2519	13,154	11,838	69	1080
Tanzania	3,297,673	27,308	89,543	809,325	5740	40,755	16,653	105	905
Uganda	2,516,788	16,350	53,503	804,079	4397	23,987	20,095	99	1249

**Fig. 2:**
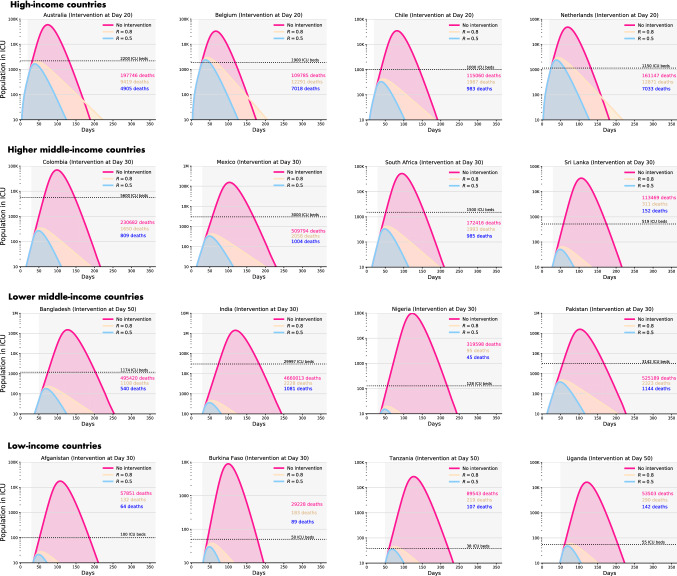
Impact of single, sustained mitigation or suppression strategy on total deaths in 16 countries over a 12-month period

*Sensitivity analyses* As sensitivity analyses, we found that a single but continuous yearlong mitigation or suppression strategy would be effective to keep the number of patients well below the available hospital capacity (Fig. 2). In case of suppression, in 3 months, most of the countries would not have any new cases to report. In case of sustained mitigation, countries would require approximately 6.5 months to reach a no-new-case scenario (Fig. 2). Additionally, dynamic mitigation and suppression interventions implemented for a period of time less than 50 days led to an increase in the number of infections beyond the ICU healthcare capacities. The same was observed for relaxation periods longer than 30 days.

## Discussion

In this mathematical modelling study, we have assessed the potential impact of dynamic community-based NPIs, involving sixteen economically diverse countries, as a pragmatic strategy for controlling the COVID-19 pandemic in order to provide a practical illustration of interventions and strategies implemented to reduce the reproduction rate of COVID-19. Our study has several inter-related findings. First, we show that simultaneous cycles of 50-day mitigation (*R* value of 0.8) followed by a 30-day relaxation could provide means to reduce the effective reproduction number, however, will be insufficient to keep the number of patients requiring ICU care within manageable levels. Second, by contrast, we found that dynamic cycles of 50-day suppression (*R* value of 0.5) followed by a 30-day relaxation would be required, for all countries, to keep ICU demands below the national capacities. Third, significant number of new infections and deaths could be prevented if these “rolling” suppression measures can be maintained for an 18-month period, or until a suitable treatment and/or vaccination become available. Finally, a continuous, yearlong suppression strategy may also reduce overall attack rates significantly and appears effective. However, implementation (and socioeconomic sustenance) of such stringent measure could be challenged by its detrimental impacts on population well-being and livelihood.

Our findings may have several explanations. First, despite higher rates of contact across older age groups [3], we predict a somewhat lower incidence of ICU hospitalisation and deaths in low-income settings. This can be explained, at least partly, by the demographic differences with a relatively younger average age structure of these populations, and absence of integrated death registration system. However, given the significant inequalities in baseline health, testing capabilities and critical care infrastructure across the countries, in reality, a higher overall level of excess deaths are likely in resource-poor settings owing to health systems failure, especially in uncontrolled or mitigation intervention scenarios. Second, it was unsurprising that a more restrictive suppression strategy (*R*: 0.5) in our study reduced ICU hospitalisations and deaths for all countries. This is because a further reduction in the reproductive number secondary to more stringent interventions can maximally reduce the population transmissibility of the SARS-CoV-2 [24]. Notably, implementation of such strategies also creates a policy dilemma for many low-income countries: how to address the “competing priorities” of preventing COVID-19 associated deaths and public health system failure with the long-term economic collapse and hardship. In this regard, we have observed that in contrast to a long fixed-duration social distancing, dynamic NPIs (that reduce the overall attack rates effectively) may offer a helpful balance.

Third, in our study, dynamic cycles of 50-day suppression followed by a 30-day relaxation were effective to lower the deaths significantly for all countries since both transmissibility and case severity (and by extension, critical care demands) were significantly reduced throughout the 18-month period. Notably, this intermittent combination of strict social distancing, and a relatively relaxed period (with efficient testing, case isolation, contact-tracing and shielding of the vulnerable), may allow populations and the national economies to “breathe” at intervals—a potential that might make this solution more sustainable, especially in resource-poor regions [25]. The specific durations of these interventions can be defined by specific countries according to their needs and local facilities, what is key is to identify a combination pattern that allows to protect the health of the population not only from COVID-19 but also from economic hardship and mental health issues. Finally, these findings reinforce the value of dynamic social distancing strategies estimated by earlier studies for the UK, Canada and China [3, 25, 26], and extend these to multiple global regions under various dynamic intervention scenarios.

The strengths and limitations of our study merit careful consideration. First, as restrictive NPIs may need to be maintained worldwide for many months, we have examined the impacts of dynamic NPIs to “switch on” and “switch off” at regular intervals. These measures have shown to be largely unaffected to uncertainties in effective *R* estimates and in the severity of the virus [4]. Second, NPI strategies only blunt (however prolong) the epidemic cycle, since there is lesser build-up of herd immunity while these interventions are kept in place. If these measures are, however, lifted altogether, a second (potentially more serious) outbreak could occur [27]. Therefore, in the absence of individual-level data and more detailed country-specific parameters, our study provides an illustrative comparison of different “rolling” strategies to suggest (a) when such measures could be lifted, and (b) for how long. Third, we used the most up-to-date disease transmission parameters [4, 17, 18, 20] to construct our adaptive models, based on well-established SEIR model of epidemic dynamics for infectious diseases. Fourth, since different interventions are likely to be implemented differentially and may have a heterogeneous effect in multiple locations, we have chosen a broad illustrative target of reducing the reproduction number *R* rather than specific community measures that may differ significantly by context. Fifth, we employed age-standardized estimates of hospitalization and infection-fatality-ratios in countries with diverse demographic structures, and considered countries at various categories of national income, in order to provide useful “context-specific” estimates. Finally, we used *rise-and-fall* timescale of infections (50 days, in the absence of intervention) as the ideal intervention duration and calculated 30-day as the optimal break duration before triggering the next cycle, however specific to each country other combinations could be considered depending the specific settings and availability of resources. In this regard, triggering dynamic interventions based on a specific pre-specified mortality number or rate, as was done in earlier modelling for the UK [3], would not be optimal for under-developed countries since (a) the health systems are less efficient to ascertain all new cases comprehensively, and (b) a younger demographic would mean that by the time the target mortality threshold is reached for the trigger, the countries have already accrued a significantly large number of cases.

Our study also had several important limitations. In the absence of country-specific, real-time, reproduction numbers for the epidemic, we assumed a constant transmission rate during each modeled cycle. These estimates are likely to vary by a population’s adherence to the NPI and the mix of specific measures put in place. In this respect, our chosen effective *R* estimates of 0.8 and 0.5 reflect two scenarios of weaker and stronger reduction in transmission, respectively, which could be achieved through social distancing measures and the interruption of transmission chains (e.g., through ramping up testing, contact tracing, isolation and quarantine and other potential strategies chosen by individual countries). We anticipate that the countries will be able to introduce additional control measures with time that might counterbalance the detrimental effect of decreasing compliance. The age-standardisation analyses were based on public sector surveillance data, which may not be robust for all LMIC and LIC countries, with potentials for underestimation of cases and deaths. Furthermore, given unavailability of relevant data, we were unable to adjust for wider social and economic costs of the dynamic approaches; further studies will be needed to quantify these aspects. Additional factors such as potential seasonal variations, environmental pollutions or structural determinants may influence, at least in part, these interventions, highlighting the need of flexibility in terms of the suitable strategy and combination of interventions that can be implemented in each country. Finally, similar to all modelling studies, our analyses were based on several transmission parameter assumptions. Since some uncertainties exist around the natural history and local transmission dynamics of the SARS-CoV-2, the precise efficacy and optimal duration of the dynamic strategies may differ for other countries and will need to be tailored accordingly.

Our study may have important implications. First, we have reported several findings relevant to COVID-19 management and policy development. We provide an actionable strategy option for COVID-19 control by employing dynamic interventions that could delay the epidemic peak, while allowing time to enhance health systems capacities and efforts to develop therapies or vaccines. These dynamic measures also allow interim periods of relaxation in order to minimise socioeconomic disruptions and maximise population compliance to these stringent suppression measures. However, these should be weighed carefully against costs, any risks imposed to the society, and the social protection available in each setting. Second, these findings also stimulate further relevant research that may involve: (a) more in-depth analyses of detailed natural history of the disease (e.g., including transmissibility in asymptomatic state) based on patient-level data, when available, from various countries [28], (b) various spatial pathways and patterns of epidemic in different circumstances (e.g., co-morbidity, reinfection) and settings (e.g., urban vs. rural); and (c) targeted modelling studies accounting for genomic susceptibility [29], social behaviour [30] and economic diversity [3].

In conclusion, this multi-country analysis demonstrates that intermittent reductions of *R* below 1 through a potential combination of suppression interventions and relaxation can be a pragmatic strategy for COVID-19 pandemic control. Such a “schedule” of social distancing might be particularly relevant to low-income countries, where a single, prolonged suppression intervention is unsustainable. As a policy option, efficient implementation of dynamic suppression interventions worldwide, therefore, would help: (1) prevent critical care overload and deaths, (2) gain time to develop preventive and clinical measures, and (3) reduce economic hardship globally.

## Electronic supplementary material

Below is the link to the electronic supplementary material.

Supplementary material 1 (PDF 84 kb)

Supplementary material 2 (PDF 182 kb)
