# Supplementary material for: Dynamic interventions to control COVID-19 pandemic: a multivariate prediction modelling study comparing 16 worldwide countries
Source: Eur J Epidemiol. 2020 May 19;35(5):389–99. doi: 10.1007/s10654-020-00649-w (PMC7237242; doi:10.1007/s10654-020-00649-w)
Supplement: Supplementary file 2 — Supplementary material 2 (PDF 182 kb) [file 10654_2020_649_MOESM2_ESM.pdf]

Table S1: Estimates of the severity of cases for Australia

Age group	N	Proportion of total population, %	Proportion of infected individuals hospitalised, %	Proportion of hospitalised cases requiring critical care, %	Proportion of individuals requiring critical care die, %	Infection fatality rate, %
0-9 years	3280238	13.02	0.00	0.00	0.00	0.00
10-19 years	3079378	12.22	0.04	0.00	0.00	0.01
20-29 years	3401525	13.50	1.04	5.00	59.42	0.03
30-39 years	3662343	14.53	3.43	5.00	49.21	0.08
40-49 years	3282597	13.02	4.25	6.30	60.13	0.16
50-59 years	3093653	12.27	8.16	12.20	59.77	0.60
60-69 years	2605017	10.34	11.80	27.40	59.69	1.93
70-79 years	1768659	7.02	16.60	43.20	59.68	4.28
80+ years	1029790	4.09	18.40	70.90	59.79	7.80
**Age-standardised rates, %**			**5.34**	**29.23**	**59.57**	**0.93**

Table S2: Estimates of the severity of cases for Belgium

Age group	N	Proportion of total population, %	Proportion of infected individuals hospitalised, %	Proportion of hospitalised cases requiring critical care, %	Proportion of individuals requiring critical care die, %	Infection fatality rate, %
0-9 years	1305219	11.31	0.00	0.00	0.00	0.00
10-19 years	1298970	11.26	0.04	0.00	0.00	0.01
20-29 years	1395385	12.09	1.04	5.00	59.42	0.03
30-39 years	1498535	12.99	3.43	5.00	49.21	0.08
40-49 years	1524152	13.21	4.25	6.30	60.13	0.16
50-59 years	1601891	13.88	8.16	12.20	59.77	0.60
60-69 years	1347696	11.68	11.80	27.40	59.69	1.93
70-79 years	908725	7.88	16.60	43.20	59.68	4.28
80+ years	658753	5.71	18.40	70.90	59.79	7.80
**Age-standardised rates, %**			**6.01**	**31.46**	**59.62**	**1.13**

Table S3: Estimates of the severity of cases for Chile

Age group	N	Proportion of total population, %	Proportion of infected individuals hospitalised, %	Proportion of hospitalised cases requiring critical care, %	Proportion of individuals requiring critical care die, %	Infection fatality rate, %
0-9 years	2450918	12.93	0.00	0.00	0.00	0.00
10-19 years	2505672	13.22	0.04	0.00	0.00	0.01
20-29 years	3020205	15.94	1.04	5.00	59.42	0.03
30-39 years	2878807	15.19	3.43	5.00	49.21	0.08
40-49 years	2556775	13.49	4.25	6.30	60.13	0.16
50-59 years	2328585	12.29	8.16	12.20	59.77	0.60
60-69 years	1737346	9.17	11.80	27.40	59.69	1.93
70-79 years	950339	5.01	16.60	43.20	59.68	4.28
80+ years	523388	2.76	18.40	70.90	59.79	7.80
**Age-standardised rates, %**			**4.69**	**25.78**	**59.51**	**0.72**

Table S4: Estimates of the severity of cases for the Netherlands

Age group	N	Proportion of total population, %	Proportion of infected individuals hospitalised, %	Proportion of hospitalised cases requiring critical care, %	Proportion of individuals requiring critical care die, %	Infection fatality rate, %
0-9 years	1762690	10.31	0.00	0.00	0.00	0.00
10-19 years	1973468	11.54	0.04	0.00	0.00	0.01
20-29 years	2106722	12.32	1.04	5.00	59.42	0.03
30-39 years	2075858	12.14	3.43	5.00	49.21	0.08
40-49 years	2201959	12.88	4.25	6.30	60.13	0.16
50-59 years	2520370	14.74	8.16	12.20	59.77	0.60
60-69 years	2109482	12.34	11.80	27.40	59.69	1.93
70-79 years	1526904	8.93	16.60	43.20	59.68	4.28
80+ years	819669	4.79	18.40	70.90	59.79	7.80
**Age-standardised rates, %**			**6.12**	**30.61**	**59.62**	**1.12**

Table S5: Estimates of the severity of cases for Colombia

Age group	N	Proportion of total population, %	Proportion of infected individuals hospitalised, %	Proportion of hospitalised cases requiring critical care, %	Proportion of individuals requiring critical care die, %	Infection fatality rate, %
0-9 years	7448799	14.80	0.00	0.00	0.00	0.00
10-19 years	8231614	16.35	0.04	0.00	0.00	0.01
20-29 years	8779218	17.44	1.04	5.00	59.42	0.03
30-39 years	7667022	15.23	3.43	5.00	49.21	0.08
40-49 years	6339173	12.59	4.25	6.30	60.13	0.16
50-59 years	5445614	10.82	8.16	12.20	59.77	0.60
60-69 years	3633308	7.22	11.80	27.40	59.69	1.93
70-79 years	1882391	3.74	16.60	43.20	59.68	4.28
80+ years	912304	1.81	18.40	70.90	59.79	7.80
**Age-standardised rates, %**			**3.93**	**23.25**	**59.44**	**0.54**

Table S6: Estimates of the severity of cases for Mexico

Age group	N	Proportion of total population, %	Proportion of infected individuals hospitalised, %	Proportion of hospitalised cases requiring critical care, %	Proportion of individuals requiring critical care die, %	Infection fatality rate, %
0-9 years	22245383	17.44	0.00	0.00	0.00	0.00
10-19 years	22356958	17.52	0.04	0.00	0.00	0.01
20-29 years	21623928	16.95	1.04	5.00	59.42	0.03
30-39 years	18636625	14.61	3.43	5.00	49.21	0.08
40-49 years	16343173	12.81	4.25	6.30	60.13	0.16
50-59 years	12397493	9.72	8.16	12.20	59.77	0.60
60-69 years	7946332	6.23	11.80	27.40	59.69	1.93
70-79 years	4023962	3.15	16.60	43.20	59.68	4.28
80+ years	2001674	1.57	18.40	70.90	59.79	7.80
**Age-standardised rates, %**			**3.57**	**22.33**	**59.41**	**0.47**

Table S7: Estimates of the severity of cases for South Africa

Age group	N	Proportion of total population, %	Proportion of infected individuals hospitalised, %	Proportion of hospitalised cases requiring critical care, %	Proportion of individuals requiring critical care die, %	Infection fatality rate, %
0-9 years	11581615	19.78	0.00	0.00	0.00	0.00
10-19 years	10240605	17.49	0.04	0.00	0.00	0.01
20-29 years	10231760	17.47	1.04	5.00	59.42	0.03
30-39 years	9942466	16.98	3.43	5.00	49.21	0.08
40-49 years	6845747	11.69	4.25	6.30	60.13	0.16
50-59 years	4794113	8.19	8.16	12.20	59.77	0.60
60-69 years	3068429	5.24	11.80	27.40	59.69	1.93
70-79 years	1430792	2.44	16.60	43.20	59.68	4.28
80+ years	422740	0.72	18.40	70.90	59.79	7.80
**Age-standardised rates, %**			**3.09**	**19.07**	**59.22**	**0.35**

Table S8: Estimates of the severity of cases for Sri Lanka

Age group	N	Proportion of total population, %	Proportion of infected individuals hospitalised, %	Proportion of hospitalised cases requiring critical care, %	Proportion of individuals requiring critical care die, %	Infection fatality rate, %
0-9 years	3383992	15.87	0.00	0.00	0.00	0.00
10-19 years	3369304	15.80	0.04	0.00	0.00	0.01
20-29 years	2906780	13.63	1.04	5.00	59.42	0.03
30-39 years	2883558	13.52	3.43	5.00	49.21	0.08
40-49 years	2848798	13.36	4.25	6.30	60.13	0.16
50-59 years	2533919	11.88	8.16	12.20	59.77	0.60
60-69 years	1966154	9.22	11.80	27.40	59.69	1.93
70-79 years	1080639	5.07	16.60	43.20	59.68	4.28
80+ years	350590	1.64	18.40	70.90	59.79	7.80
**Age-standardised rates, %**			**4.38**	**24.20**	**59.50**	**0.63**

Table S9: Estimates of the severity of cases for Bangladesh

Age group	N	Proportion of total population, %	Proportion of infected individuals hospitalised, %	Proportion of hospitalised cases requiring critical care, %	Proportion of individuals requiring critical care die, %	Infection fatality rate, %
0-9 years	29140694	17.87	0.00	0.00	0.00	0.00
10-19 years	30882112	18.94	0.04	0.00	0.00	0.01
20-29 years	29600040	18.15	1.04	5.00	59.42	0.03
30-39 years	26177061	16.05	3.43	5.00	49.21	0.08
40-49 years	20143207	12.35	4.25	6.30	60.13	0.16
50-59 years	14480320	8.88	8.16	12.20	59.77	0.60
60-69 years	6892779	4.23	11.80	27.40	59.69	1.93
70-79 years	4064814	2.49	16.60	43.20	59.68	4.28
80+ years	1665146	1.02	18.40	70.90	59.79	7.80
**Age-standardised rates, %**			**3.10**	**19.60**	**59.27**	**0.36**

Table S10: Estimates of the severity of cases for India

Age group	N	Proportion of total population, %	Proportion of infected individuals hospitalised, %	Proportion of hospitalised cases requiring critical care, %	Proportion of individuals requiring critical care die, %	Infection fatality rate, %
0-9 years	236731829	17.32	0.00	0.00	0.00	0.00
10-19 years	252674336	18.49	0.04	0.00	0.00	0.01
20-29 years	238481457	17.45	1.04	5.00	59.42	0.03
30-39 years	212399683	15.54	3.43	5.00	49.21	0.08
40-49 years	165881490	12.14	4.25	6.30	60.13	0.16
50-59 years	125378954	9.18	8.16	12.20	59.77	0.60
60-69 years	84296275	6.17	11.80	27.40	59.69	1.93
70-79 years	37500685	2.74	16.60	43.20	59.68	4.28
80+ years	13073046	0.96	18.40	70.90	59.79	7.80
**Age-standardised rates, %**			**3.35**	**20.34**	**59.32**	**0.41**

Table S11: Estimates of the severity of cases for Nigeria

Age group	N	Proportion of total population, %	Proportion of infected individuals hospitalised, %	Proportion of hospitalised cases requiring critical care, %	Proportion of individuals requiring critical care die, %	Infection fatality rate, %
0-9 years	62691322	31.20	0.00	0.00	0.00	0.00
10-19 years	46319357	23.05	0.04	0.00	0.00	0.01
20-29 years	32244205	16.04	1.04	5.00	59.42	0.03
30-39 years	23840172	11.86	3.43	5.00	49.21	0.08
40-49 years	16454206	8.19	4.25	6.30	60.13	0.16
50-59 years	10366004	5.16	8.16	12.20	59.77	0.60
60-69 years	6059156	3.02	11.80	27.40	59.69	1.93
70-79 years	2555573	1.27	16.60	43.20	59.68	4.28
80+ years	433608	0.22	18.40	70.90	59.79	7.80
**Age-standardised rates, %**			**1.96**	**16.28**	**59.07**	**0.19**

Table S12: Estimates of the severity of cases for Pakistan

Age group	N	Proportion of total population, %	Proportion of infected individuals hospitalised, %	Proportion of hospitalised cases requiring critical care, %	Proportion of individuals requiring critical care die, %	Infection fatality rate, %
0-9 years	52774521	24.37	0.00	0.00	0.00	0.00
10-19 years	44914765	20.74	0.04	0.00	0.00	0.01
20-29 years	39377474	18.18	1.04	5.00	59.42	0.03
30-39 years	29843795	13.78	3.43	5.00	49.21	0.08
40-49 years	20586127	9.51	4.25	6.30	60.13	0.16
50-59 years	14690100	6.78	8.16	12.20	59.77	0.60
60-69 years	8500213	3.93	11.80	27.40	59.69	1.93
70-79 years	4464790	2.06	16.60	43.20	59.68	4.28
80+ years	1413532	0.65	18.40	70.90	59.79	7.80
**Age-standardised rates, %**			**2.55**	**19.03**	**59.23**	**0.29**

Table S13: Estimates of the severity of cases for Afghanistan

Age group	N	Proportion of total population, %	Proportion of infected individuals hospitalised, %	Proportion of hospitalised cases requiring critical care, %	Proportion of individuals requiring critical care die, %	Infection fatality rate, %
0-9 years	11040694	29.02	0.00	0.00	0.00	0.00
10-19 years	9635671	25.33	0.04	0.00	0.00	0.01
20-29 years	6779023	17.82	1.04	5.00	59.42	0.03
30-39 years	4381488	11.52	3.43	5.00	49.21	0.08
40-49 years	2846500	7.48	4.25	6.30	60.13	0.16
50-59 years	1773768	4.66	8.16	12.20	59.77	0.60
60-69 years	1020779	2.68	11.80	27.40	59.69	1.93
70-79 years	458747	1.21	16.60	43.20	59.68	4.28
80+ years	105087	0.28	18.40	70.90	59.79	7.80
**Age-standardised rates, %**			**1.86**	**16.41**	**59.05**	**0.18**

Table S14: Estimates of the severity of cases for Burkina Faso

Age group	N	Proportion of total population, %	Proportion of infected individuals hospitalised, %	Proportion of hospitalised cases requiring critical care, %	Proportion of individuals requiring critical care die, %	Infection fatality rate, %
0-9 years	6431030	31.65	0.00	0.00	0.00	0.00
10-19 years	4879742	24.01	0.04	0.00	0.00	0.01
20-29 years	3397789	16.72	1.04	5.00	59.42	0.03
30-39 years	2356070	11.59	3.43	5.00	49.21	0.08
40-49 years	1524531	7.50	4.25	6.30	60.13	0.16
50-59 years	939519	4.62	8.16	12.20	59.77	0.60
60-69 years	516654	2.54	11.80	27.40	59.69	1.93
70-79 years	227339	1.12	16.60	43.20	59.68	4.28
80+ years	48708	0.24	18.40	70.90	59.79	7.80
**Age-standardised rates, %**			**1.81**	**15.96**	**59.01**	**0.17**

Table S15: Estimates of the severity of cases for Tanzania

Age group	N	Proportion of total population, %	Proportion of infected individuals hospitalised, %	Proportion of hospitalised cases requiring critical care, %	Proportion of individuals requiring critical care die, %	Infection fatality rate, %
0-9 years	17990384	31.01	0.00	0.00	0.00	0.00
10-19 years	13636144	23.51	0.04	0.00	0.00	0.01
20-29 years	9575102	16.51	1.04	5.00	59.42	0.03
30-39 years	6938129	11.96	3.43	5.00	49.21	0.08
40-49 years	4635689	7.99	4.25	6.30	60.13	0.16
50-59 years	2803032	4.83	8.16	12.20	59.77	0.60
60-69 years	1556334	2.68	11.80	27.40	59.69	1.93
70-79 years	710015	1.22	16.60	43.20	59.68	4.28
80+ years	160632	0.28	18.40	70.90	59.79	7.80
**Age-standardised rates, %**			**1.90**	**16.31**	**59.04**	**0.18**

Table S16: Estimates of the severity of cases for Uganda

Age group	N	Proportion of total population, %	Proportion of infected individuals hospitalised, %	Proportion of hospitalised cases requiring critical care, %	Proportion of individuals requiring critical care die, %	Infection fatality rate, %
0-9 years	14582039	32.94	0.00	0.00	0.00	0.00
10-19 years	11067913	25.00	0.04	0.00	0.00	0.01
20-29 years	7564888	17.09	1.04	5.00	59.42	0.03
30-39 years	4881270	11.03	3.43	5.00	49.21	0.08
40-49 years	2997016	6.77	4.25	6.30	60.13	0.16
50-59 years	1765499	3.99	8.16	12.20	59.77	0.60
60-69 years	930221	2.10	11.80	27.40	59.69	1.93
70-79 years	391414	0.88	16.60	43.20	59.68	4.28
80+ years	89327	0.20	18.40	70.90	59.79	7.80
**Age-standardised rates, %**			**1.61**	**15.10**	**58.92**	**0.15**
